# Prognostic significance of bi/oligoclonality in childhood acute lymphoblastic leukemia as determined by polymerase chain reaction

**DOI:** 10.1590/S1516-31802001000500005

**Published:** 2001-09-01

**Authors:** Carlos Alberto Scrideli, Ricardo Defavery, José Eduardo Bernardes, Luíz Gonzaga Tone

**Keywords:** Childhood acute lymphoblastic leukemia, Polymerase chain reaction, Oligoclonality, Leucemia linfóide aguda da infância, Reação em cadeia da polimerase, Oligoclonalidade

## Abstract

**CONTEXT:**

The CDR-3 region of heavy-chain immunoglobulin has been used as a clonal marker in the study of minimal residual disease in children with acute lymphoblastic leukemia. Southern blot and polymerase chain reaction studies have demonstrated the occurrence of bi/oligoclonality in a variable number of cases of B-lineage acute lymphoblastic leukemia, a fact that may strongly interfere with the detection of minimal residual disease. Oligoclonality has also been associated with a poorer prognosis and a higher chance of relapse.

**OBJECTIVES:**

To correlate bi/oligoclonality, detected by polymerase chain reaction in Brazilian children with B-lineage acute lymphoblastic leukemia with a chance of relapse, with immunophenotype, risk group, and disease-free survival.

**DESIGN:**

Prospective study of patients’ outcome.

**SETTING:**

Pediatric Oncology Unit of the University Hospital, Faculty of Medicine of Ribeirão Preto, University of São Paulo.

**PARTICIPANTS:**

47 children with acute lymphoblastic leukemia

**DIAGNOSTIC TEST:**

Polymerase chain reaction using consensus primers for the CDR-3 region of heavy chain immunoglobulin (FR3A, LJH and VLJH) for the detection of clonality.

**RESULTS:**

Bi/oligoclonality was detected in 15 patients (31.9%). There was no significant difference between the groups with monoclonality and biclonality in terms of the occurrence of a relapse (28.1% versus 26.1%), presence of CALLA+ (81.2% versus 80%) or risk group (62.5% versus 60%). Disease-free survival was similar in both groups, with no significant difference (p: 0.7695).

**CONCLUSIONS:**

We conclude that bi/oligoclonality was not associated with the factors investigated in the present study and that its detection in 31.9% of the patients may be important for the study and monitoring of minimal residual disease.

## RESUMO

**INTRODUÇÃO:**

A região CDR-3 da IgH têm sido usada com marcador clonal no estudo de doença residual mínima em crianças com leucemia linfóide aguda. Estudos por Southern blot e reação em cadeia da polimerase têm demonstrado bi/oligoclonalidade em um número variável de casos de leucemia linfóide aguda de linhagem B, o que pode interferir de forma importante na detecção de doença residual mínima. Oligoclonalidade também têm sido associada com pior prognóstico e maior chance de recidiva.

**OBJETIVOS:**

Correlacionar bi/oligoclonalidade detectada por reação em cadeia da polimerase em crianças brasileiras portadoras de leucemia linfóide aguda de linhagem B com chance de recaída, imunofenótipo, grupo de risco e sobrevida livre de doença.

**TIPO DE ESTUDO:**

Estudo prospectivo de seguimento de pacientes.

**LOCAL:**

Serviço de Oncologia Pediátrica do Hospital das Clínicas da Faculdade de Medicina de Ribeirão Preto- USP.

**PARTICIPANTES:**

47 crianças portadoras de leucemia linfóide aguda de linhagem B

**TESTE DIAGNÓSTICO:**

Reação em cadeia da polimerase, utilizando-se primers de consenso para região CDR-3 da IgH (FR3A, LJH e VLJH) para detecção de clonalidade.

**RESULTADOS:**

Bi/oligoclonalidade foi encontrada em 15 pacientes (31,9%). Não houve diferença estatística significativa entre os grupos com monoclonalidade e biclonalidade quanto a presença de recidiva (28,1% versus 26,1%), presença de CALLA+ (81,2% versus 80%) ou grupo de risco (62,5% versus 60%). Sobrevida livre de doença foi similar em ambos os grupos, sem diferença significativa (p: 0,7695).

**CONCLUSÕES:**

Concluímos que bi/oligoclonalidade não esteve associada com fatores os analisados neste estudo e que sua detecção em 31,9% dos pacientes pode ser importante no estudo e seguimento de doença residual mínima

## INTRODUCTION

During the differentiation of B-lineage lymphocytes, recombinations in the variability (V), diversity (D) and junction (J) segments occur in the heavy-chain immunoglobulin (IgH) gene, resulting in relatively conserved regions (frameworks) and hypervariable regions (complementarity determining regions, CDRs). One of these hypervariable regions, CDR-3, is unique in each B-cell lineage.^1–3^ These sequences can be amplified by polymerase chain reaction using consensus primers for the conserved regions that flank the CDR-3 of IgH^4–7^ and can be used as clonal markers of B-lineage acute lymphocytic leukemia in minimal residual disease studies.^3,8–16^

The presence of oligoclonal populations in B-lineage acute lymphoblastic leukemia has been detected in a variable number of cases in studies by Southern blot and polymerase chain reaction and may be associated with a poorer prognosis for the disease.^17–19^ Furthermore, the presence of more than one clone detected at diagnosis may strongly interfere with the detection of minimal residual disease.^20–23^

In the present study we correlated the presence of bi/oligoclonality, detected by polymerase chain reaction in Brazilian children with B-lineage acute lymphoblastic leukemia, with immunophenotype, risk group and disease-free survival.

## METHODS

### Patients

Sixty pediatric patients with B-lineage acute lymphoblastic leukemia were admitted for treatment to the Pediatric Clinic of the University Hospital, Faculty of Medicine of Ribeirão Preto, University of São Paulo, from December 1990 to September 1996. Forty-seven of these children were eligible for the study, 11 were excluded due to lack of stored DNA at diagnosis or lack of polymerase chain reaction amplification, and 2 were lost to follow-up. The diagnosis was based on morphological analysis according to the criteria proposed by the French-American-British cooperative group,^24^ and on immunophenotyping by flow cytometry with monoclonal antibodies. The patients were classified and treated according the Brazilian Childhood Leukemia Treatment Group (GBTLI) protocols.^25^ The 47 patients studied (14 girls and 33 boys) ranged in age from 7 months to 13 years (mean: 5.3 years). Complete remission was considered to have occurred when morphological analysis showed less than 5% blasts in bone marrow, and was obtained within 45-117 months (median: 83 months). The patients were investigated in terms of immunophenotyping, risk group and event-free survival.

Of the 47 patients studied, 38 were classified as common leukemia (CALLA+), five as early pre-B (CALLA-), one as B (slg+), one as biphenotypic (lymphoid/myeloid), one as B-lineage lymphoid blast transformation (CALLA-) involving a patient with chronic myelogenous leukemia, and in one it was not possible to perform immunotyping, although amplification for IgH was obtained by polymerase chain reaction. Twenty-nine children were considered to be at high risk for relapse and 18 were considered to be at standard risk, in accordance with the Brazilian Childhood Leukemia Treatment Group criteria.^25^ Karyotype obtained by bone marrow aspirates was analyzed by G-banding techniques, according to ISCN criteria. Some of these patients were reported in a previous study.^7^

### Sample Preparation and Polymerase Chain Reaction

Bone marrow samples were obtained by aspiratory puncture at diagnosis and analyzed by polymerase chain reaction in accordance with Saiki et al.^26^ DNA was extracted by digestion with proteinase K, extraction with phenol/chloroform/isoamyl alcohol, precipitation with sodium acetate and ethanol, and quantification by spectrophotometry at 260 and 280 nm absorbance.^27^

Genomic DNA (0.1-0.2 μg) was added to 23 μl of reaction solution containing 2 mM of each dNTP, 2.5 μl reaction buffer (Gibco BRL, Gaithersburg, MD, USA), 1.5 mM MgCl_2_, 1 U taq polymerase, 0.5 to 1 μl FR3A primer (sense), LJH or VLJH (antisense),^5^ and 20 μl mineral oil. After an initial denaturation at 94 °C for 5 minutes and annealing at 57 °C for 2 minutes, each sample was submitted to 35 cycles with extension at 72 °C, denaturation at 94 °C and annealing at 54 °C for 1 minute 20 seconds each, with final extension at 72 °C for 10 minutes in a RoboCycler 40 thermocycler (Stratagene, La Jolla, CA, USA). Care was taken to reduce the risk of sample contamination.^28^ A positive and negative control was used for each reaction and all samples were analyzed at least twice.

**Figure 1:**
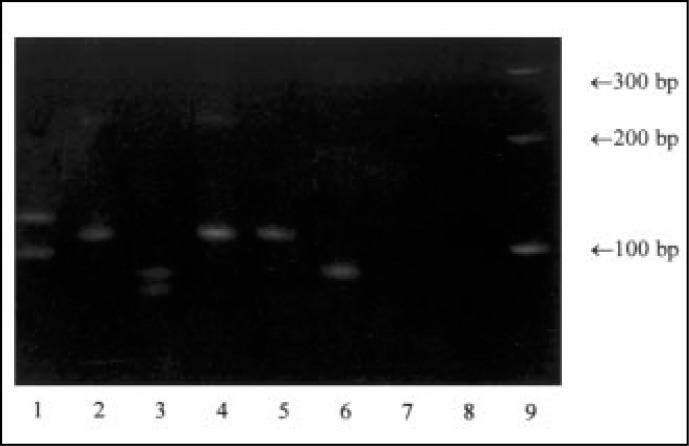
Polymerase chain reaction amplification using CDR-3 primers and DNA from acute lymphoblastic leukemia patients with bi/oligoclonal (lanes 1 and 3), monoclonal (lanes 2, 4, 5 and 6) and negative controls with (lane 7) and without DNA (lane 8). Lane 9 represents molecular weight markers.

A 10-15 μl amount of the product amplified by polymerase chain reaction in 5 μl ficoll/bromophenol blue buffer was submitted to 15% polyacrylamide gel electrophoresis (1-8 V/cm), stained with ethidium bromide (1 μg/ml), visualized in a ultraviolet transilluminator and photographed.^27^ A marker with fragments of known size was used for comparison with the samples during electrophoresis. The clonality of B-lineage lymphocytes was characterized by the presence of one fragment (monoclonal) or 2 or more fragments (bi/oligoclonal) of homogeneous size from 80 to 120 pb^4,5,10^ (Figure 1).

Data were analyzed statistically by the exact Fisher test for mean comparison, and disease-free survival was analyzed by Kaplan-Meyer survival analysis and by the log-rank test,^29^ with a cut-off date in July 2000. The disease-free survival of each child was analyzed, with the event being time of relapse or death. Patients who died without reaching remission were counted as having the event during month zero. The calculations were made using the GraphPad Prism software (San Diego, CA, USA).

## RESULTS

Bi/oligoclonality was detected in 15 of the 47 patients studied (31.9%) and monoclonality in 32 (68.1%). Of the 32 children in the monoclonality group, 9 (28.1%) suffered a relapse (9 high-risk, 5 CALLA+), 20 (62.5%) were classified as being at high risk for a relapse (9 relapsed, 15 CALLA+) and 26 (81.2%) presented CALLA+ (5 relapsed, 15 high-risk). Of the 15 children in the bi/oligoclonality group, 4 (26.6%) suffered a relapse or did not present clinical remission (4 high-risk, 3 CALLA+), 9 (60%) were classified as being at high risk for a relapse (4 relapsed, 6 CALLA+), and 12 (80%) had CALLA+ (3 relapsed, 6 high-risk). G-banding cytogenetic study was made in 20/47 patients, as shown in Table 2.

Of the 15 patients with bi/oligoclonality, 7 were submitted to karyotyping analysis. Changes were found in 5 cases: 2 with changes of the chromosome 14, with trisomy in one of them and structural alteration [t(2;14)(p12;q31)] in the other; and 3 patients presented monosomy of sex chromosomes (2 in the X chromosome and one in the Y).

Of the 32 patients with monoclonality, 13 were submitted to karyotyping analysis. Structural changes were the most frequent alterations, consisting of 5 translocations [1 t(2;17)(p22;q25), 1 t(8;14)(q24;q31), 2 t(4;11)(q21;q25) and 1 (9;22)(q32;q11)] and one deletion [del 16(q22)]. All of these patients suffered relapses or died. Near triploid/hyperdiploidy was observed in 2 patients who were in remission (Table 1).

**Table 1:** Patients with bi/oligoclonality according to age, sex, immunophenotype, karyotype, IgH-polymerase chain reaction and clinical outcome

*Patient*	*Age/Sex*	*Immunophenotype*	*Karyotype/Risk.group*	*CDR-3*	*Clinical outcome -PCR (months)*
*L1*	*10y/F*	*CALLA/SR*	*45, X, -X [2], 46, X, -X, +21q +[9] 40-45, X, -X, +21q +[cp17]*	*++*	*CCR (+117)*
*L9*	*9y/M*	*CALLA/SR*	*46, XY [6], 44-48, XY, +8, [cp2]*	*+*	*CCR (+116)*
*L13*	*11y/M*	*CALLA/HR*	***NM***	*++*	*CCR (+116)*
*L14*	*11y/M*	*CALLA/HR*	*53-54, XY, +X, +5, +6, +10, +11, +14, +17, +18, +21, +mar [cp9]*	*++*	*CCR (+115)*
*L35*	*7m/M*	*CALLA/HR*	*46, XY [14], 43-45, XY, −20 [cp3]*	*+*	*Relapse (+47)*
*L37*	*12y/M*	*CALLA/HR*	*47, XY, t(2;14)(p12;q31), +mar [10] 36-48, XY, −14, +17, t(2;14)(p12;q31) [cp23]*	*++*	*Noremission*
*L43*	*1y/M*	*CALLA/HR*	***NM***	*+*	*Relapse (+10)*
*L44*	*8y/M*	*CML blast phase/ early pre-B/HR*	*t(9;22)(q32;q11)*	*+*	*Relapse (+03)*
*L53*	*3y/M*	*CALLA/SR*	***NM***	*+*	*CCR (+110)*
*L72*	*3y/F*	*CALLA/SR*	*46, XX, [4], 46, X, -X, +9q- [5] 37-46, X, -X, −15, +9q-, −18, −20, −22 [cp7]*	*++*	*CCR (+105)*
*L77*	*2y/M*	*CALLA/HR*	*46, XY, [1] 46, XY, t(2;17)(p22;q25) [5] 41-46, XY, t(2;17)(p22;q25) [cp10]*	*+*	*Accidental death (+18)*
*L85*	*4y/M*	*CALLA/HR*	*57-67 [16](hypo/near triploid)*	*+*	*CCR (+105)*
*L86*	*9y/M*	*CALLA/SR*	*46, XY [1] 46, XY, +7, −21 [3], 62-68[10] (near triploid)*	*+*	*CCR (+104)*
*L90*	*2y/M*	*CALLA/SR*	*46, XY[15] 39-43, XY, −13, −16, −19 [cp4]*	*+*	*CCR (+104)*
*L97*	*7y/M*	*B (sIg +)/HR*	*46, XX[2] 40-48, XY, +2, dup(7)(q21;q31), −8, t(8;14)(q24;q31), +19, +20, +21, +22, +mar [17]*	*+*	*No remission*
*L111*	*8m/M*	*early pre-B/HR*	*46, XY [2] 44-46, XY, t(4;11)(q21;q25) [cp4]*	*+*	*Relapse (+31)*
*L122*	*3y/F*	*CALLA/SR*	*46, XX [1], 45-54, XX, +15 [cp2]*	*++*	*CCR (+96)*
*L126*	*2y/M*	*CALLA/SR*	*46, XY [7], 38-39, XY, −8, −19, −20, +21, +22,+mar [cp9]*	*++*	*Death-infection (+34)*
*L133*	*6y/M*	*CALLA/SR*	***NM***	*+*	*CCR (+96)*
*L137*	*5y/M*	*early pre-B/HR*	***NM***	*+*	*Relapse (+13)*
*L142*	*4y/F*	*CALLA/HR*	***NM***	*+*	*CCR (+95)*
*L147*	*8m/F*	*early pre-B/HR*	*46, XX [3], 46, XX, t(4;11)(q21;q25) [5] 43-46, XX, t(4;11)(q21;q25) [cp9]*	*+*	*Relapse(+08)*
*L171*	*7y/M*	*CALLA/HR*	***NM***	*+*	*CCR (+84)*
*L174*	*13y/F*	*ND/HR*	***NM***	*+*	*CCR (+84)*
*L176*	*4y/M*	*CALLA/HR*	*46, XY [1], 46, XY, del(16)(q22) [2] 43-46, XY, del(16)(q22), −9, −11, −14[cp5]*	*+*	*Relapse (+15)*
*L178*	*4y/M*	*CALLA/HR*	***NM***	*++*	*Relapse (+55)*
*L179*	*11y/M*	*CALLA/HR*	*46, XY [3]*	*+*	*CCR (+83)*
*L180*	*7y/M*	*CALLA/HR*	***NM***	*+*	*Relapse (+50)*
*L181*	*9y/M*	*CALLA/SR*	***NM***	*+*	*CCR (+83)*
*L183*	*1y/M*	*early-preB/HR*	***NM***	*++*	*CCR (+82)*
*L192*	*2y/M*	*CALLA/HR*	*46, XY [2], 37-46, X, -Y [cp3]*	*++*	*Death-sepsis (+3)*
*L194*	*4y/F*	*CALLA/HR*	*46, XX [2]39-46, XX, −9, −16/16p-, +19, −22 [cp7]*	*+*	*CCR (+79)*
*L213*	*5y/M*	*CALLA/HR*	***NM***	*+*	*CCR (+76)*
*L214*	*8y/M*	*CALLA/SR*	***NM***	*+*	*CCR (+76)*
*L223*	*5y/F*	*CALLA/SR*	***NM***	*+*	*CCR (+74)*
*L237*	*3y/F*	*CALLA/HR*	***NM***	*+*	*Relapse (+02)*
*L244*	*3y/M*	*CALLA/HR*	***NM***	*++*	*Relapse (+38)*
*L263*	*6y/F*	*CALLA/SR*	***NM***	*+*	*Death-sepsis (+14)*
*L310*	*5y/M*	*CALLA/SR*	***NM***	*++*	*CCR (+61)*
*L339*	*4y/F*	*CALLA/SR*	***NM***	*+*	*CCR (+56)*
*L360*	*9y/M*	*CALLA/HR*	***NM***	*+*	*CCR (+54)*
*L369*	*6y/M*	*CALLA/HR*	***NM***	*+*	*CCR (+53)*
*L380*	*3y/M*	*biphenotypic/HR*	***NM***	*++*	*CCR (+52)*
*L389*	*2y/F*	*early pre-B/HR*	***NM***	*++*	*Relapse (+9)*
*L417*	*7y/M*	*CALLA/SR*	***NM***	*+++*	*CCR (+46)*
*L428*	*11y/F*	*CALLA/HR*	***NM***	*+*	*CCR (+45)*
*L432*	*6y/F*	*CALLA/HR*	***NM***	*+*	*CCR (+45)*

***Abbreviations: F -female, M-male, CCR - complete continuous remission, CALLA - common leukemia (CD 10+), CML - chronic myelogenous leukemia, NM - no metaphase available, HR - high risk, SR - standard risk, + one band identified, ++ two bands identified, +++ three bands identifie***

**Figure 2:**
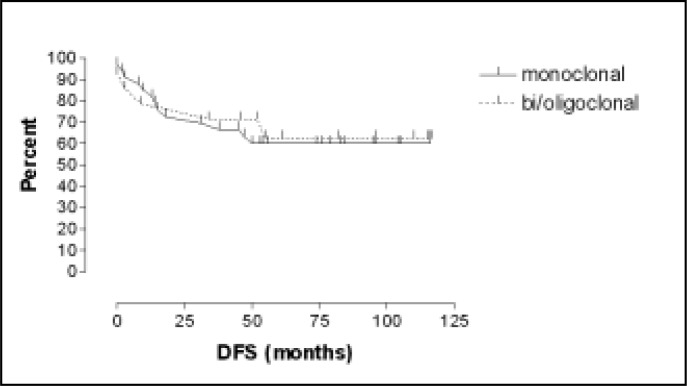
Kaplan-Meier plots of disease-free survival (DFS) for patients with monoclonal and bi/oligoclonal IgH gene rearrangements.

There was no significant difference between groups in terms of relapse (p: 1.000), risk group (p: 1.000) or presence of CALLA (p: 1.000) when the data were analyzed by the exact Fisher test. Disease-free survival was also similar for both groups (p: 0.7695), with no significant difference by the log-rank survival method (Table 2 and Figure 2). The complete continuous remission times for the groups were similar, 47-117 months (median: 96) for the bi/oligoclonal group and 45-116 months (median: 83) for the monoclonal group.

**Table 2:** Relapse, risk group and CALLA presence among the monoclonal and bi/oligoclonal groups

	*Relapse*	*high risk*	*CALLA +*
***monoclonal***	*9/32 (28.1%)*	*20/32 (62.5%)*	*26/32 (81.2%)*
***bi/oligoclonal***	*4/15 (26.6%)*	*9/15 (60.0%)*	*12/15 (80.0%)*

## DISCUSSION

Appropriate leukemia classification is essential for improving therapeutic approaches. Several factors have been clearly associated with higher chances of relapse, while others are currently under investigation. Age, white blood cell count at diagnosis, immunophenotyping, DNA index, cyto-reduction time, central nervous system involvement and specific chromosomal abnormalities have been shown to be useful as prognostic factors and have been used for classifying childhood acute lymphoblastic leukemia in risk groups, with differentiated treatment protocols for each group.^25^

Some studies^17–19^ have associated the presence of oligoclonality in childhood Blineage acute lymphoblastic leukemia with an adverse clinical outcome, which could have a use as a prognostic factor. This association, however, has not detected by others.^20,35,36,42,43^ In the present study, when patients with bi/oligoclonality and monoclonality were compared in terms of relapse, presence of CALLA and risk group, no statistically significant differences were detected. Analysis of disease-free survival has also shown no difference between the two groups up to the present time and, in our patients, this does not suggest that bi/oligoclonality was associated with worse clinical outcome.

Despite improvements in leukemia treatment, 20-30% of children still relapse.^12,17^ The study of minimal residual disease for follow-up and early detection of relapses using consensus primers for CDR-3 has been used by several authors for establishing prognosis in such patients.^8,9,11,12,39,40^ The presence of bi/oligoclonality at diagnosis, as well as clonal evolution during the course of the disease, may be a problem in the detection and study of minimal residual disease using primers or probes for rearranged VH-D-JH. This is due to the possibility that smaller clones present at diagnosis may emerge as major clones in acute lymphoblastic leukemia patients who suffer a relapse.^20,21,34^ It has been suggested that the instability of IgH rearrangements increases as a function of time.^33,38^ The presence of bi/oligoclonality or clonal evolution, although relatively frequent, is mostly associated with the same D-JH sequence, with events in the rearranged VH gene being more common (VH to VH, VH to D-JH).^30,33,37,38,41^

Bi/oligoclonality in a rearranged IgH gene has been detected in 20 to 50% of B-lineage acute lymphoblastic leukemia cases in studies by Southern blot^17,18,30–32^ and in 10-40% of cases by polymerase chain reaction.^20,31,33,34^ The explanation for the difference in oligoclonality findings may be due to the fact that incomplete D-J rearrangements can be detected by southern blot but not by polymerase chain reaction. The latter is normally based on the use of primers for the V region that may not be present in such rearrangements.^31,33^ In the present study, bi/oligoclonality was detected by polymerase chain reaction in 31.9% of cases.

Some mechanisms have been proposed for explaining the presence of bi/oligoclonality in B-lineage acute lymphoblastic leukemia. The IgH gene located on chromosome 14q32.2^31^ may be amplified in the presence of chromosome 14 polysomy. Kitchingman et al.^18^ detected hyperdiploidy in 9/18 pediatric patients with oligoclonality, and 8 of them presented polysomy of chromosome 14. Forestier et al.,^35^ Moreira et al.^36^ and Schardt et al.,^30^ in studies on children and adults, respectively, found no alterations of chromosome 14. In the present study, the karyotype was analyzed in 7/15 cases with bi/oligoclonality, and changes in chromosome 14 were detected in 2 cases, one of them with polysomy and the other with structural alterations, i.e. t(2;14)(p12;q31). These data suggest that other mechanisms in addition to polysomy of chromosome 14 may be involved.

Another explanation for bi/oligoclonality may be the presence of two different cell populations in bone marrow, either due to two separate events or, more commonly, to the formation of subclones. Four different mechanisms have been proposed thus far for the formation of subclones: VH-VH substitution, VH rearrangement in a preexisting D-JH segment, substitution in the rearranged DJH gene, or ongoing rearrangements in a non-rearranged precursor cell.^32,37,38^ Some authors^17–19^ suggest that these clones might be associated with unfavorable clinical outcome, because they could be responsible for disease progression, through selection of those with higher proliferation rate and acquired drug resistance. This study and others^20,35,36,42,43^ are in disagreement with that hypothesis, since there was no association between the presence of oligoclonality and adverse clinical outcome.

## CONCLUSION

The presence of bi/oligoclonality was not associated with a greater chance of relapse, immunophenotyping or risk group, and that its detection in 31.9% of the patients may be important for the study and follow-up of minimal residual disease.

## References

[1] Tonegawa S (1983). Somatic generation of antibody diversity. Nature.

[2] Van Dongen JJM, Wolvers-Tettero ILM (1991). Analysis of immunoglobulin and T-cell receptor genes. Clin Chim Acta.

[3] Yamada M, Hudson S, Tournay O (1989). Detection of minimal residual disease in hematopoietic malignancies of B-cell lineage using third-complementarity-determining region (CDR III) specific probes. Proc Natl Acad Sci USA.

[4] Trainor KJ, Brisco MJ, Story CJ, Morley AA (1990). Monoclonality in B-lymphoproliferative disorders detected at the DNA level. Blood.

[5] Trainor KJ, Brisco MJ, Wan JH, Neoh S, Grist S, Morley AA (1991). Gene rearrangement in B- and T-lymphoproliferative disease detected by the polymerase chain reaction. Blood.

[6] Lehman CM, Sarago C, Nasin S (1995). Comparison of PCR with southern hybridization for the routine detection of immunoglobulin heavy-chain gene rearrangements. Am J Clin Pathol.

[7] Scrideli CA, Simões AL, Defavery R, Bernardes JE, Duarte MHO, Tone LG (1997). Childhood B-lineage acute lymphoblastic leukemia clonality study by the polymerase chain reaction. J Pediatr Hematol/Oncol.

[8] Potter MN, Steward CG, Oakhill A (1993). The significance of detection of minimal residual disease in childhood acute lymphoblastic leukemia. Br J Haematol.

[9] Yokota S, Hansen-Hagge TE, Ludwig WD (1991). Use of polymerase chain reaction to monitor minimal residual disease in acute lymphoblastic leukemia patients. Blood.

[10] Brisco MJ, Tan LN, Orsborn AN, Morley AA (1990). Development of a highly sensitive assay based on the polymerase chain reaction for rare B-lymphocyte clones in a polyclonal population. Br J Haematol.

[11] Brisco MJ, Condon J, Hughes E (1994). Outcome prediction in childhood acute lymphoblastic leukemia by molecular quantification of residual disease at end of induction. Lancet.

[12] Kuang SQ, Gu LJ, Dong S (1996). Long-term follow-up of minimal residual disease in childhood acute lymphoblastic leukemia patients by polymerase chain reaction analysis of multiple clone-specific of malignancy-specific gene markers. Cancer Genet Cytogenet.

[13] Cavé H, ten Bosch JW, Suciu S (1998). Clinical significance of minimal residual disease in childhood acute lymphoblastic leukemia. N Engl J Med.

[14] Grhun B, Hongeng S, Yi H (1998). Minimal residual disease after intensive induction therapy in childhood acute lymphoblastic leukemia predicts outcome. Leukemia.

[15] Goulden NJ, Knechtli CJS, Garland RJ (1998). Minimal residual disease analysis for the prediction of relapse in children with standard-risk acute lymphoblastic leukemia. Br J Haematol.

[16] Foroni L, Harrison C, Hoffbrand AC, Potter MN (1999). Investigation of minimal residual disease in childhood and adult acute lymphoblastic leukemia by molecular analysis. Br J Haematol.

[17] Beishuizen A, Verhoeven MAJ, Van Wering ER, Hahlen K, Hooijkass H, van Dongen JJM (1994). Analysis of Ig and T-cell receptor genes in 40 childhood acute lymphoblastic leukemias at diagnosis and subsequent relapse: implications for the detection of minimal residual disease by polymerase chain reaction analysis. Blood.

[18] Kitchingman G, Mirro J, Stass S (1986). Biological and prognostic significance of the presence of more than two m heavy-chain genes in childhood acute lymphoblastic leukemia of B-precursor cell origin. Blood.

[19] Green E, McConvile CM, Powell JE (1998). Clonal diversity of Ig and T-cell-receptor gene rearrangements identifies a subset of childhood B-precursor acute lymphoblastic leukemia with increased risk of relapse. Blood.

[20] Coyle LA, Papaioannou M, Yaxley JC (1996). Molecular analysis of leukaemic B cells in adult and childhood acute lymphoblastic leukemia. Br J Haematol.

[21] Baruchel A, Cayuela JM, MacIntyre E (1995). Assessment of clonal evolution at Ig/TCR loci of leukemia by single-strand conformation polymorphism studies and high resolutive PCR derived methods: implication for a general strategy of minimal residual disease detection. Br J Haematol.

[22] Van Dongen JJM, Seriu T, Panzer-Grumayer ER (1998). Prognostic value of minimal residual disease in acute lymphoblastic leukemia in childhood. Lancet.

[23] Szczenpanski T, Beishuizen A, Pongers-Willemse MJ (1999). Cross-lineage T-cell receptor gene rearrangements in more than ninety percent of childhood precursor-B acute lymphoblastic leukemias: alternative PCR targets for detection of minimal residual disease. Leukemia.

[24] Bennett J, Catovsky O, Daniel M (1976). French-American-British (FAB) cooperative group proposals for the classification of acute leukemias. Br J Haematol.

[25] Brandalise S, Odone V, Pereira W, Andrea M, Zanichelli M, Aranega A (1993). Treatment results of three consecutive Brazilian cooperative childhood ALL protocols: GBTLI-80, GBTLI-82 and −85. ALL Brazilian Group. Leukemia.

[26] Saiki RK, Gelfand DH, Stoffel S (1988). Primer-directed enzymatic amplification of DNA with a thermostable DNA polymerase. Science.

[27] Sambrook J, Fritsch EF, Maniatis J (1989). Molecular Cloning - A Laboratory Manual.

[28] Kwok S, Higuchi R (1989). Avoiding false positives with PCR. Nature.

[29] Peto R, Pike MC, Armitage P (1977). Design and analysis of randomized clinical trials requiring prolonged observation of each patient. Br J Cancer.

[30] Schardt C, Hoelzer D, Ganser A (1992). Presence of more than two rearranged immunoglobulin heavy-chain genes in adult precursor B-cell acute lymphoblastic leukemia. Ann Hematol.

[31] Height SE, Swansbury GJ, Matutes E, Treleaven JG, Catovsky D, Dyer MJS (1996). Analysis of clonal rearrangements of Ig heavy chain locus in acute leukemia. Blood.

[32] Choi Y, Greenberg SJ, Du TL (1996). Clonal evolution in Blineage acute lymphoblastic leukemia by contemporaneous VHVH gene replacements and VH-DJH gene rearrangements. Blood.

[33] Steward CG, Goulden NJ, Katz F (1994). A polymerase chain reaction study of the stability of Ig heavy-chain and T-cell receptor d gene rearrangements between presentation and relapse of childhood B-lineage acute lymphoblastic leukemia. Blood.

[34] Campana D, Van Dongen JJM, Pui CH, Pui CH (1999). Minimal residual disease. Childhood leukemias.

[35] Forestier E, Nodenson I, Lindström A (1994). Simultaneous immunoglobulin T-cell receptor gene rearrangements and multiclonality in childhood acute lymphoblastic leukemia. Acta Paediatr.

[36] Moreira I, Papaioannou M, Palmisano GL (1998). B-cell oligoclonality in ALL: a mixed bag of IgH clone with important biological, clinical and prognostic significance. Blood.

[37] Steenbergen EJ, Verhagen OJHM, van Leeuwen EF, den Borne AEGK, van der Schoot E (1993). Distinct ongoing Ig heavy-chain rearrangement process in childhood B-precursor acute lymphoblastic leukemia. Blood.

[38] Wasserman R, Yamada M, Ito Y (1992). VH gene rearrangement events can modify the immunoglobulin heavy chain during progression of B-lineage acute lymphoblastic leukemia. Blood.

[39] Yamada M, Wasserman R, Lange B, Reichard BA, Womer RB, Rovera G (1990). Minimal residual disease in childhood Blineage lymphoblastic leukemia: persistence of leukemic cells during the first 18 months of treatment. N Engl J Med.

[40] Maeda Y, Horiuchi F, Morita S (1994). Determination of minimal residual disease using clone-specific primers for CDR III in patients with acute lymphoblastic leukemia with or without Philadelphia chromosome: possibility of clinical application as a tool for improving prognosis. Experimental Hematol.

[41] Rovera G, Wasserman R, Yamada M (1991). Detection of minimal residual disease in childhood leukemia with the polymerase chain reaction. New Engl J Med.

[42] Katz F, Ball L, Gibbons B, Chessels J (1989). The use of DNA probes to monitor minimal residual disease in acute lymphoblastic leukemia. Br J Haematol.

[43] Ghali DW, Panzer S, Fisher S (1995). Heterogeneity of T-cell receptor d gene indicating subclone formation in acute precursor B-cell leukemias. Blood.

